# A Giant Brunneroma Causing Gastrointestinal Bleeding and Severe Anemia Requiring Transfusion and Surgery

**DOI:** 10.1155/2017/6940649

**Published:** 2017-02-19

**Authors:** Nicola C. Frenkel, Miangela M. Laclé, Inne H. M. Borel Rinkes, Izaak Q. Molenaar, Jeroen Hagendoorn

**Affiliations:** ^1^Department of Surgery, University Medical Center Utrecht, Utrecht, Netherlands; ^2^Department of Pathology, University Medical Center Utrecht, Utrecht, Netherlands

## Abstract

Brunner's gland hamartoma, also called hyperplasia, adenoma, and Brunneroma, is an extremely rare benign proliferative lesion of Brunner's glands in the duodenum. While being mostly small and asymptomatic, they can result in gastrointestinal bleeding and obstruction. We report the case of a 54-year-old man presenting with melena and severe anemia requiring blood transfusion. CT scans showed a large mass of 8 cm in diameter, presumably arising in the duodenum. Endoscopic biopsies were not conclusive. As we were unable to determine the nature of the mass preoperatively and due to the severe symptoms, its size, and the uncertain malignant potential, a classic Whipple procedure was performed. The resected specimen showed extensive proliferation of Brunner's glands without signs of malignancy.

## 1. Introduction

Brunner's glands were first described in 1688 by Johann Brunner, a Swiss anatomist. Brunner's glands are localized in the submucosal layer of the duodenum, predominantly in the most proximal part, and decrease in prevalence in the second and third parts of the duodenum. Brunner's glands secrete an alkaline fluid that protects the epithelial lining of the duodenum from the acid chime of the stomach [1–3]. Brunner's gland adenoma was first described by Curveilheir in 1835. It is generally described as an extremely rare benign hamartomatous lesion characterized by the proliferation of Brunner's glands [1, 4, 5]. Over the years, it has been referred to as Brunner's gland adenoma, hamartoma, hyperplasia, and Brunneroma. In most cases, they are an accidental finding as they are rarely larger than 2 cm and often asymptomatic [1, 3, 6]. We report a rare case in which a large Brunner's gland hamartoma presented with melena and severe anemia requiring blood transfusion and on which a classic Whipple procedure was performed.

## 2. Case Report

A 54-year-old Caucasian man presented with melena since four days. A single episode of melena had occurred a year before but had not warranted further investigation. Presently, the melena did not resolve and the patient started to develop symptoms of nausea, vomiting (no hematemesis), and lack of appetite. Moreover, the patient had an increasing sense of lightheadedness and fatigue. Four days after the start of these symptoms, the patient experienced severe fatigue and the continued sensation of near-fainting. The patient then contacted the hospital where he was admitted. Physical examination showed an anemic man in weakened condition, tachycardia (pulse 110 beats/minute), low normal blood pressure of 116/61 mmHg, and otherwise normal vital signs (temperature 37.4°C, pulse oximetry saturation 100%). Except confirmation of the melena during rectal examination, no further abnormalities were found during physical examination. Hematological investigations showed a severe normocytic anemia with 2.6 mmol/L hemoglobin (normal range, 8.6–10.7 mmol/L). The patient received a total of 5 transfusions of erythrocyte concentrates, after which the hemoglobin level remained stable at 6.0 mmol/L. Computed tomography analysis showed a lobular mass of around 8 cm near the proximal duodenum. Ultrasound investigation showed a solid mass with smooth borders, partly vascularized. It was difficult to identify whether or not the mass originated from the intestine itself. Endoscopic investigation revealed a large swelling immediately distal to the pylorus, covered with apparently normal mucosa. It was not possible to see the entirety of the mass due to its size. An origin of the intestinal bleeding was not observed. However, during biopsies, the mass showed a quick inclination to bleed. Biopsies were not conclusive.

**Figure 1:**
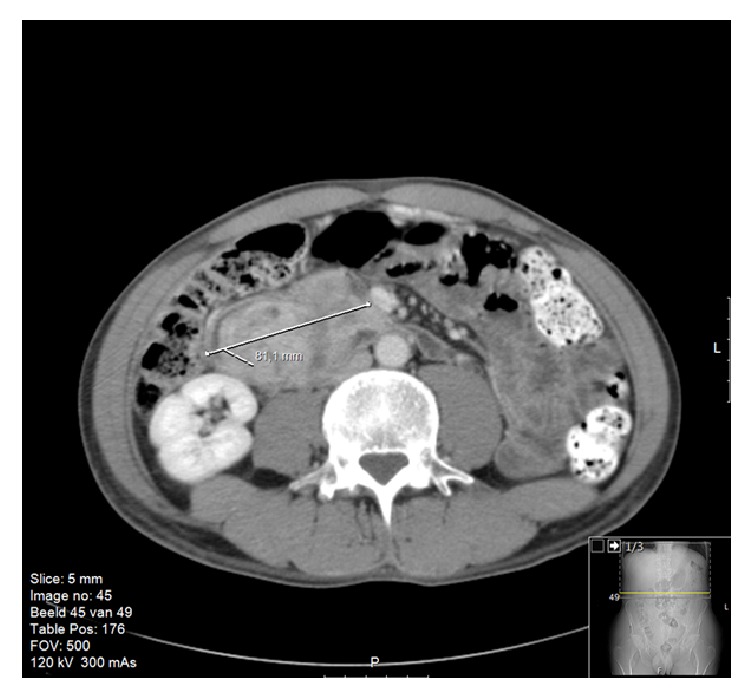
Axial CT view showing a large mass of approximately 8 cm in diameter, near the proximal duodenum. Oral and IV contrast were given.

**Figure 2:**
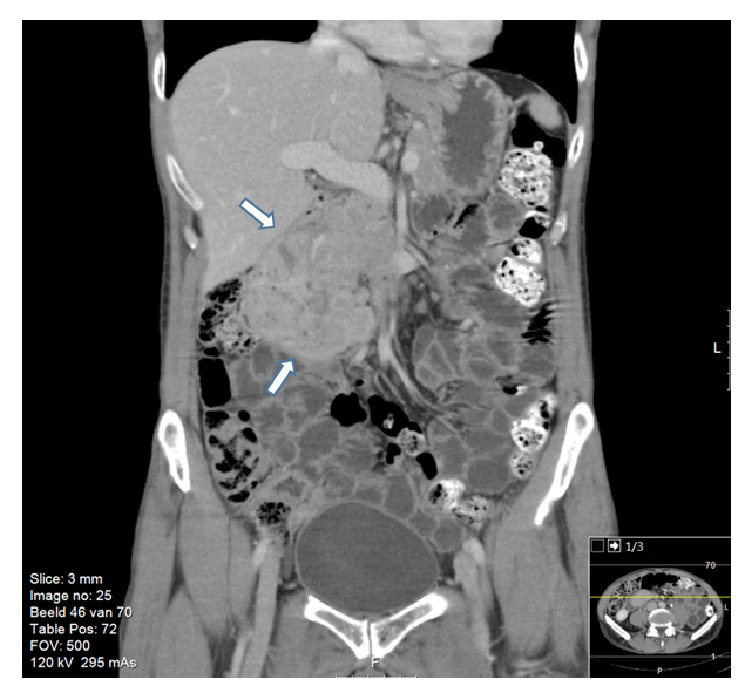
Coronal CT view showing a large mass near the proximal duodenum. Oral and IV contrast were given.

During multidisciplinary discussion, it was decided that the most likely diagnosis was a gastrointestinal stromal cell tumor (GIST). Given the severe anemia, risk of acute bleeding, the tumor's size, and uncertain malignant potential, urgent surgical intervention was deemed necessary. The patient was then transferred to our hospital for further examination and presurgical workup for a possible Whipple procedure. The CT scan was repeated, which confirmed a large (8 cm diameter) hypervascular exophytic mass (Figures 1 and 2). It appeared to originate from the descending duodenum. There were no signs of invasion or dissemination.

**Figure 3:**
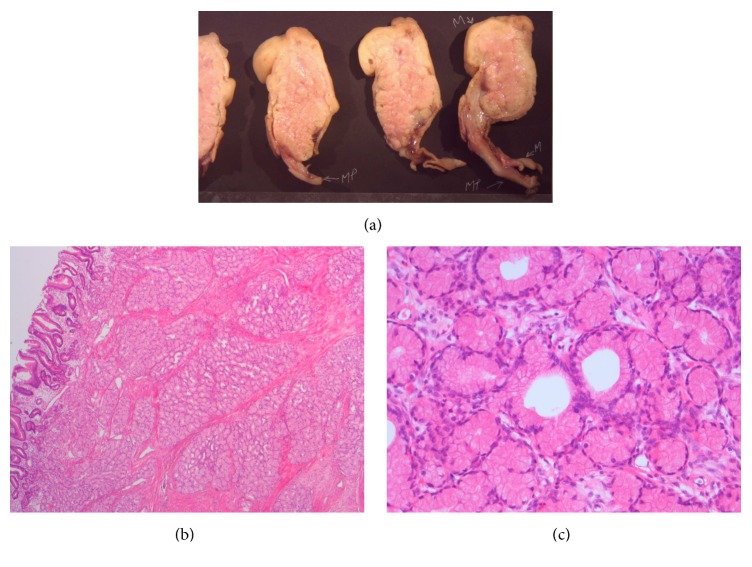
Histopathological examination. (a) 8 × 4 × 8 cm submucosal polypoid tumor in the proximal duodenum. ((b) and (c)) Microscopic examination showed extensive proliferation of Brunner's glands.

An uncomplicated classical Whipple procedure was performed. Gross macroscopic examination of the resection specimen showed an 8 × 4 × 8 cm submucosal polypoid tumor located in the proximal duodenum. A cut section showed a firm tan tumor with a small lobular pattern. The polypoid tumor was located 5 cm from the pancreas. Microscopically, the lesion showed marked proliferation of Brunner's glands, extending up towards the antrum of the stomach (Figures 3(a), 3(b), and 3(c)). Moreover, the mucosa of the duodenum showed extensive foveolar metaplasia as well as focal ectopic gastric mucosa. There were no signs of malignancy.

Several days into the postoperative period, the intra-abdominal drain near the wound bed showed chylous fluid drainage. The patient was placed on a medium-chain triglyceride diet but was switched to total parenteral nutrition when his oral intake was determined to be insufficient. The abdominal drain was removed on day 13 after surgery. The patient's oral intake increased and the total parenteral nutrition was stopped. The patient was discharged on day 17. The patient recovered well and had no recurring symptoms the following year. To our knowledge, never before has a Brunner's gland hamartoma been described presenting with such severe anemia and this size, requiring blood transfusions and a Whipple procedure. A previously documented case of severe anemia due to Brunner's gland hamartoma was reported to present with similar symptoms of lightheadedness and melena and a hemoglobin level of 61 g/L (3.8 mmol/L). This case also required erythrocyte concentrate transfusions and as the size was relatively small (3 cm) a segmental duodenal resection was performed [7].

## 3. Discussion

The nomenclature surrounding this lesion has been a point of discussion over the years. The terms Brunner's gland hamartoma, Brunner's gland hyperplasia, Brunner's gland proliferative lesion, and Brunneroma have been used interchangeably. In general, they all refer to a benign hamartomatous proliferating lesion of Brunner's glands. Kim et al. distinguish between hyperplasia and hamartoma [8]. They describe Brunner's gland hyperplasia as multiple small lesions of excessive numbers of Brunner's gland and Brunner's gland hamartoma as a solitary lesion consisting of Brunner's glands, ducts, smooth muscle, adipose tissue, and lymphoid cells [8]. As the distinction is considered arbitrary, many pathologists follow these guidelines: a lesion less than 5 mm both solitary or multiple is described as hyperplasia. A lesion greater than 5 mm is called a hamartoma [9]. The use of the term “adenoma” has been opposed as more typical neoplastic architectural and cytological features are not usually observed within these lesions. Instead, they appear to be normal-looking Brunner's glands showing a nodular proliferation [8, 9].

The prevalence of Brunner's gland hamartoma remains unclear since they are generally discovered incidentally and their rarity does not allow for definitive studies. Brunner's gland hamartomas usually present in the fifth or sixth decades of life and appear to have no gender or race preference. Benign lesions of the small intestine in themselves are a rarity and Brunner's gland hamartoma constitutes a small part of these lesions [1–3]. Botsford et al. observed one Brunner's gland adenoma in a study of 115 primary tumors of the small intestine [10]. In a study of 615 benign and malignant duodenal specimens, 2.5% constituted Brunner's gland hyperplasia and 1.4% Brunner's gland hamartoma [11].

Most often, at discovery, this type of lesion presents as a single pedunculated polyp reaching an average size of 1-2 cm. Extremely rare are the so-called giant Brunner's gland hamartomas, reportedly reaching sizes of around 10 cm [3, 6, 12]. As most Brunner's gland hamartomas are small (<2 cm) and asymptomatic, the cases in which endoscopic or surgical intervention is required are very rare. This mostly concerns the hamartomas that are large in size and symptomatic. Symptomatic cases most often present with gastrointestinal bleeding or obstructive symptoms [1, 2, 6, 7, 9, 12–14]. In rare cases, Brunner's gland hamartomas have been reported to cause duodenal intussusception [15, 16], “idiopathic” pancreatitis by intermittent obstruction of the Ampulla of Vater [17, 18], and mimicking of pancreatic or duodenal malignancy [15, 19, 20]. In Table 1, an overview of cases with Brunner's gland hamartomas >5 cm is discussed. As patients usually present with alarming symptoms and endoscopic and imaging investigations show a large abdominal mass, knowledge on whether the mass is benign or malignant is crucial for deciding on further steps. However, as Brunner's gland hamartomas are submucosal, endoscopic biopsies are often not conclusive and therefore cannot aid in the decision-making process [3, 4]. Therefore, a comprehensive differential diagnosis is imperative, including leiomyoma, leiomyosarcoma, adenocarcinoma, lymphoma, gastrointestinal stromal cell tumors, carcinoid tumors, heterotopic pancreatic or gastric tissue, and metastatic tumors [4, 9]. As Table 1 shows, in cases of large Brunneromas, the chosen surgical interventions often include transduodenal excision or even more drastic approaches such as a partial gastrectomy, duodenectomy, or even pancreaticoduodenectomy. These complex surgeries carry a risk of serious morbidity while the mass is eventually discovered to be a benign Brunneroma lesion. However, when biopsies are inconclusive and malignancy is suspected, in all likelihood such complex surgical interventions remain the most prudent course of action. Even when biopsies are conclusive and a Brunner's gland hamartoma is diagnosed, discussion remains on whether removal is necessary. Although Brunner's gland hamartomas are generally perceived as benign lesions, several cases of malignancy have been reported [20–24]. In a study of 722 Brunner gland's hyperplasia specimens, Sakurai et al. observed that 2.1% showed dysplastic changes, of which 0.3% showed invasive carcinoma [25]. There was no apparent correlation between the grade of atypia and the size of the lesion. Therefore, while some advocate a conservative approach due to the commonly benign nature and often small size of the lesion, the fact that malignant transformation is possible causes others to support therapeutic intervention. When possible, endoscopic polypectomy or mucosal resection is advised, while for larger tumors surgical resection should be carefully considered, bearing in mind the higher risk of complications [3, 6, 9, 16, 18, 19]. In our patient's case, due to its large size, alarming symptoms, and the possibility of a gastrointestinal stromal tumor, a surgical approach consisting of a classic pancreaticoduodenectomy was decided on.

The etiology and pathogenesis of Brunner's gland hamartoma remains unclear. Since Brunner's gland secretions are proposed to provide antiacid protection, it has been proposed that hyperacidic conditions could stimulate the formation of Brunner's gland hyperplasia. However, this seems unlikely since the use of antiacid inhibitors appears to have no effect on the lesions [1]. Furthermore, chronic* Helicobacter pylori* infection has been proposed to play a pathogenic role as several reports have observed their coexistence while others could find no significant correlation. The fact that* H. pylori* infection is so highly prevalent in the general population and Brunner's gland hamartomas are extremely rare also does not support this as the main pathogenic cause [12, 25, 26]. Recently, Akaki et al. have suggested that duodenal mucosal damage could be associated with Brunner's gland proliferation [12]. They found that high proliferative areas of Brunner's glands were situated beneath epithelium showing surface erosion and gastric foveolar metaplasia [12]. The formation of gastric foveolar metaplasia is frequently associated with duodenal ulceration and has been postulated to form as part of a mucosal repair process. Interestingly, gastric metaplasia has also been observed in conditions of duodenal hyperacidity [27]. Our resected specimen showed extensive gastric metaplasia, consistent with peptic duodenitis. Moreover, areas of focal ectopic gastric mucosa were observed, a possible cause of increased acidity and the peptic duodenitis. The latter has been proposed to be a congenital anomaly [11]. Our patient has no history of related symptoms or antiacid medication. While the precise etiology of Brunner's gland lesions is unclear, it is likely that the presence of ectopic gastric mucosa, with the possible concomitant peptic duodenitis, is related to the development of Brunner's gland hamartoma in our patient.

## 4. Conclusion

Our patient presented with melena and severe anemia, requiring blood transfusion. To our knowledge, a Brunneroma resulting in such a severe anemia has not been described before. After analysis showed a large mass in presumably the duodenum and we were unable to determine its nature with biopsies, a classic Whipple procedure was performed. The resected specimen showed a large Brunner's gland hamartoma of 8 × 4 × 8 cm. Histopathologic examination also showed ectopic gastric mucosa in the duodenum as well as gastric metaplasia, a sign of possible peptic duodenitis. While the etiology of Brunner's gland hamartoma remains unclear, damage to the duodenal mucosa has been suggested as a possible pathologic mechanism. In our patient's case, the presence of ectopic gastric mucosa most likely resulted in increased acidity and peptic duodenitis, causing duodenal mucosa damage that likely leads to the proliferation of Brunner's glands.

## Figures and Tables

**Table 1:** Reported cases of Brunner's gland hamartomas larger than 5 cm and concomitant interventional approaches.

Reference	Size (cm)	Clinical presentation	Clinical intervention
[28]	5.5 × 3.3 × 2.2	Melena, dizziness, weakness	Duodenotomy
[18]	5.5 × 4.2 × 4.3	Recurrent pancreatitis	Pyloroduodenotomy
[29]	5.5	Upper abdominal pain	Duodenocephalopancreatectomy
[30]	5-6	Jaundice, abdominal pain	Pancreaticoduodenectomy
[15]	5 × 6	Melena, diarrhea, epigastric discomfort, weight loss, anemia	Pyloroduodenotomy
[31]	6	Anemia	Duodenotomy
[32]	6	Screen for gastric cancer	Endoscopic polypectomy
[33]	6 × 2.4	Melena, weakness, palpitations	Excisional pyloroduodenoplasty
[34]	6.0 × 0.4 × 0.2	Melena	Endoscopic polypectomy
[35]	6 × 3	Epigastric pain, nausea, vomiting, melena	Transduodenal polypectomy
[36]	6 × 3.5	Upper abdominal pain	Laparoscopic and endoscopic tumor resection
[37]	6 × 4	Weakness, anemia	Laparoscopic transduodenal polypectomy
[12]	6.4 × 3	Melena, anemia	Distal gastrectomy with lymph node dissection
[19]	6 × 5 × 3	Upper abdominal pain, nausea, weight loss	Transduodenal polypectomy
[38]	6.5 × 4 ×4	Abdominal pain	Endoscopic polypectomy
[39]	6.6 × 4.5	Epigastric pain, nausea, vomiting, weight loss	Pancreaticoduodenectomy
[17]	7	Abdominal pain, melena	Endoscopic polypectomy
[40]	7.3 × 3.4 × 2.9	Melena	Transduodenal polypectomy and pyloroplasty
[41]	7.3 × 3.4 × 2.9	Melena	Pancreaticoduodenectomy
[42]	7.5 × 6.5 × 6.5	Epigastric pain	Partial duodenectomy
[16]	7.9	Upper abdominal pain, vomiting, weight loss	Distal gastrectomy and Billroth II reconstruction
[43]	8	Abdominal pain, weight loss, melena	Transduodenal polypectomy
[5]	8 × 10	Upper abdominal pain, vomiting	Pancreaticoduodenectomy
[44]	10	Fatigue, melena	Polypectomy (surgical approach not described)
[2]	10 × 2 × 1.5	Melena, dizziness, dyspnea on exertion	Transduodenal polypectomy
[45]	10.5	Referred for further evaluation of a polypoid lesion in the duodenum	Endoscopic polypectomy
[4]	10.5	Epigastric abdominal pain, reflux symptoms	Pancreaticoduodenectomy
[46]	10 × 6 × 8	Vomiting, melena	Distal gastrectomy and partial duodenectomy
[47]	10–12	Weakness, fatigue, melena	Laparoscopic transduodenal polypectomy and pyloroplasty
[48]	12 × 10 × 8	Vomiting, weakness, weight loss	Duodenotomy and partial gastrectomy
